# Evaluation of the degradation of two bioabsorbable interference screws: an *in-vivo* study in sheep

**DOI:** 10.1590/acb370405

**Published:** 2022-06-27

**Authors:** Paulo Sérgio Scorsato, Sheila Canevese Rahal, Tania Mary Cestari, Maria Jaqueline Mamprim, Danuta Pulz Doiche, Daniel de Bortoli Teixeira, Rafael Cerântola Siqueira, Marcílio Felix

**Affiliations:** 1 PhD. Universidade de Marília – Faculty of Veterinary Medicine – Department of Veterinary Surgery and Anesthesiology – Marilia (SP), Brazil.; 2 PhD. Universidade Estadual Paulista “Júlio de Mesquita Filho” – School of Veterinary Medicine and Animal Science – Department of Veterinary Surgery and Animal Reproduction – Botucatu (SP), Brazil.; 3 PhD. Universidade de São Paulo – Bauru School of Dentistry – Department of Biological Sciences – Bauru (SP), Brazil.; 4 PhD. Universidade de Marília – Department of Agronomic Engineering – Marília (SP), Brazil.; 5 PhD. Universidade de Marília – Veterinary Medicine School – Marília (SP), Brazil.

**Keywords:** Polylactic Acid-Polyglycolic Acid Copolymer, Absorbable Implants, Bone Screws, Sheep

## Abstract

**Purpose:**

To evaluate *in-vivo* degradation of two bioabsorbable interference screws.

**Methods:**

Twenty-two crossbred Santa Inês ewes were used. A poly-DL-lactide (PDLLA) screw (70%/30%) was inserted in the right pelvic limb, and a PDLLA screw (70%) + β-tri-calcium phosphate (β-TCP) (30%) in the left pelvic limb. Animals were euthanized at one, four, seven and a half and 18 months after surgery. Plain radiography, computed tomography (CT), microCT, and histological analysis were accomplished.

**Results:**

PDLLA screw was hypodense at all evaluation moments, but with progressive density increase along the central axis, whereas PDLLA/β-TCP was initially hyperdense and progressively lost this characteristic. No adverse reactions were observed on histological evaluation.

**Conclusions:**

The inclusion of β-TCP favors screw degradation since the PDLLA/β-TCP screws evidenced a more intense degradation process than the PDLLA screws at the last evaluation. PDLLA screws showed higher bone production, evident around the screw thread, inside the lateral perforations, and in the central canal, whereas the PDLLA/β-TCP screws presented less bone tissue at the implantation site.

## Introduction

Interference screws are used in arthroscopic surgery and sports medicine in human patients to provide pressure adjustment between bone, graft/tendon and screw^1^, being mainly used for anterior cruciate ligament reconstruction^2^. Metals such as stainless steel and titanium have been used for manufacturing interference screws. However, around the 1990s, non-metallic screws were developed, which facilitate postoperative imaging studies and reduce stress by gradually shifting the load during the degradation process and, consequently, decreasing the graft damage^3^. The resistance to failure of biodegradable screws has been described as comparable to metal screws and capable of withstanding a rehabilitation program^4^^-^ 7.

Bioabsorbable screws are manufactured from various materials, including poly-DL-lactide (PDLLA), poly-L-lactide (PLLA), polylactic acid (PLA), and polyglycolic acid (PGA), or their copolymers^1^^,^ 2 ^,^ 4. There are differences in the properties of the absorbable material, such as biocompatibility, degradation kinetics, and mechanical properties^6^. Therefore, the materials can be divided into three groups:

- Group 1 has slow degradation and high crystallinity, such as poly (L-lactide), and poly (L-co-D,L-lactide) stereopolymers which have a low amount of D, L;
- Group 2 has a total degradation in one or two years, for instance, amorphous poly (L-co-D, L-lactide) stereocopolymers with a large amount of D,L;
- Group 3 has a rapid degradation rate with strength retention maintained only for several weeks, like poly-(D, L-lactide-co-glycolide) or polyglycolide-co-trimethylenecarbonate^3^.

In addition, in order to improve quality, composite materials have been developed in which inorganic particulate, such as hydroxyapatite, have been mixed into a degradable polymer matrix aiming to increase bone growth, while polymer degrades, instead of leaving a cavity^8^. On the other hand, the association of beta-tricalcium phosphate (β-TCP) with PLLA generates a more amorphous compound that shows a faster degradation^9^.

Thus, this study aimed to evaluate the degradation of two bioabsorbable interference screws composed of PDLLA or PDLLA/β-TCP, implanted in the distal femoral metaphyseal region of adult sheep, by using imaging and histology evaluations. The hypothesis was that the addition of β-TCP may favor the biomaterial degradation process.

## Methods

### Animal selection

This study was approved by the Ethics Committee on Animal Use of the Universidade de Marília (no. 27/2012).

Twenty-two healthy crossbred Santa Inês ewes, aged between 3 and 5 years old, non-pregnant females weighing from 31 to 51 kg (mean ± standard deviation–SD, 42.3 kg ± 9 kg), were used. The animals were randomly divided according to the follow*-*up and time of euthanasia, as follows:

- One month: n = 6;
- Four months: n = 5;
- Seven and a half: n = 6;
- 18 months: n = 5.

During the 10-day postoperative period, the sheep were kept in collective pens. They were fed commercial sheep feed, corn silage and Tifton hay, and provided with water *ad libitum*. After this period, the sheep were kept in a paddock with Tifton.

### Implants

**Figure 1:**
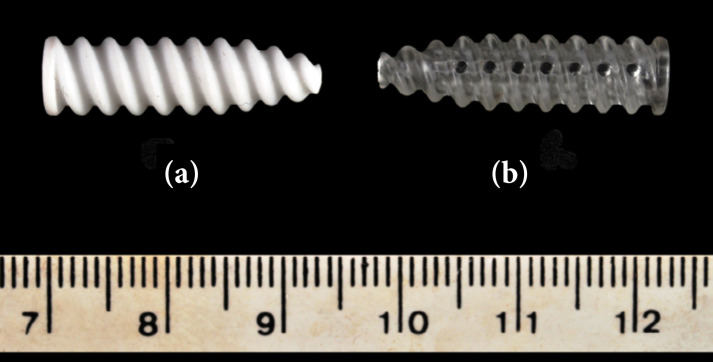
Bioabsorbable interference screws composed of **(a)** poly-D, L-lactic acid (PDLLA) with beta-tricalcium phosphate (PDLLA/β-TCP), and **(b)** poly-D, L-lactic acid (PDLLA). Note the absence of holes in **(a)** the PDLLA/β-TCP screw and seven longitudinally distributed axial holes in **(b)** the PDLLA screw.

Forty-four bioabsorbable interference screws (7 mm in diameter, 25 mm in length), sterilized by ethylene oxide, were used, 22 screws being PDLLA (70% L-lactide and 30% DL-lactide) and 22 being PDLLA/β-TCP (70/30) [(70% L-lactide and 30% D,L-lactide) (70%) + β-TCP beta-tricalcium phosphate (30%)] (Fig. 1) (Sinfix^®^; Sintegra Surgical Sciences, Pompéia, SP, Brazil). The screws had a double-entry thread, a conical-shaped distal end, a body composed of an external thread with blunt profile threads, and a central cannula for fitting the wrench. The PDLLA/β-TCP screw had no holes, but the PDLLA screw had seven axial holes distributed longitudinally every 120º.

### Surgical procedure

After 12 hours of fasting, the surgical procedures were performed with the animals under general inhalant anesthesia, combined with epidural anesthesia, and analgesia with opioids. After clipping the hair of the pelvic limbs, the sheep were placed in dorsal recumbency. Under sterile conditions, a craniolateral skin incision was made from the level of the patella to the tibial tuberosity. The fascia lata was incised, and the biceps femoris and vastus lateralis muscles were retracted to expose the distal femur. Bone perforation was performed at the level of the distal femoral metaphysis, using a 6-mm drill. After drilling, the screw was inserted with a wrench that fit inside the entire length of the screw. The wrench was designed specifically to distribute the tension force in the central canal of the screw and avoid its breakage. In addition, the wrench had a six-pointed star-shaped to combine with the central canal of the screw. In all sheep, the right pelvic limb received a PDLLA screw, and the left pelvic limb received a PDLLA/β-TCP screw. Fascia and subcutaneous tissues and skin were closed routinely using a 2–0 nylon monofilament suture.

### Pre- and post-operative care

Immediately before surgical procedure and postoperatively, penicillin in combination with streptomycin was totaling three applications. Tramadol hydrochloride was administered for four days. Cardiorespiratory parameters, body temperature, and signs of lameness were assessed once per day for 10 days after surgery. Wounds were cleaned twice daily. Skin sutures were removed 10 days after surgery. After this period, the animals were periodically inspected in the paddocks until euthanasia.

### Euthanasia

Ewes were euthanized at one, four, seven and a half and 18 months (n = 5) after surgery, for imaging studies and histological evaluations. The pelvic limbs were removed to undergo radiographic and computed tomography examinations, and after these the screw insertion region was collected and fixed in a 10% buffered formalin solution for microCT evaluation, and finally the histological examination.

### Imaging studies

Three evaluators separately assessed the right and left femurs in plain radiography, immediately after surgery and at one, four, seven and a half and 18 months postoperatively. The radiographs were taken in the craniocaudal and mediolateral positions, using Toshiba equipment, with an exposure of 52 kV and 100 mAs and a focus-film distance of 40 cm.

Cross-sectional CT images were acquired of the femur using a spiral scanner (Shimadzu SCT-7800CT; Kyoto, Japan). The scanning parameters were 120 kVp, 160 mA, with a slice thickness of 2 mm, pitch of 2 and 2 s/rotation. Images were reconstructed in multiplanar reformation using eFilm Workstation™. The quantitative evaluation consisted of measuring the density of the implant through a transverse section of the femur and a longitudinal section of the screws, with an elliptical region of interest (ROI) of approximately 0.3 cm^2^ in two measurement areas, one on the lateral surface of the screw (entrance) and the other on the medial surface of the implant (opposite to the entrance). The mean and SD of the Hounsfield unit values were obtained.

MicroCT was performed using a SkyScan 1176 X-ray microtomography (Skyscan; Kontich, Belgium). The X-ray beam source (Cone-Beam) was operated with a resolution of 80 kV, 300 uA and 12.45 µm. A Cu + Al filter was used, and the sample was rotated 360º, with a rotation step of 0.5º, generating an acquisition time of 27 minutes per sample. The number of frames executed for a final average was three in each rotation step. The two-dimensional images were reconstructed using the NRecon v.1.6.8 software (Bruker microCT; Kontich, Belgium). The DataViewer software was used to align the images and obtain coronal, sagittal and transaxial sections of the central region of the implant. Three-dimensional images were obtained using 64-bit CTVox v.2.4.0 r870 software (Bruker microCT; Kontich, Belgium).

### Histological processing

After microCT, the samples were washed in running water for 24 h and immersed in an EDTA solution (4.13% Tritriplex III and 0.44 sodium hydroxide) at the temperature from 2 to 8°C, for approximately 80 days. After demineralization, the samples were washed in running water for 12 h and immersed in chloroform for 24 h to remove the screws. Then, the samples were processed for inclusion in Histosec, obtaining alternate slices of 5 µm thick (100 µm spacing), which were stained with hematoxylin-eosin (H&E) according to the standard procedure. Photomicrographs of the implant regions were obtained with a photomicroscope consisting of an Axioskop microscope (Zeiss), AxioCam HRc digital camera and AxioVision software.

### Statistical analysis

The Hounsfield unit values obtained on CT were submitted to two-way analysis of variance (ANOVA) to compare the effect of the two screw models in relation to the implant permanence time. The Shapiro-Wilk normality test and the Bartlett’s test for homogeneity of variance data were applied, followed by the Tukey’s test for comparison of means. For all statistical analyses, two-tailed evaluations were considered with a significance level of 5%. Statistical analysis was performed using the R software.

## Results

### Trans and postoperative assessments

There were no intraoperative complications. The screws did not suffer breakage or any distortion during insertion. The sheep were monitored daily until the stitches were removed after 10 days. They did not show systemic clinical changes, and the surgical wounds healed properly, without edema, bleeding, or infection. In the paddocks, the ewes were monitored and did not show any changes until the time of euthanasia.

### Radiographic exams

**Figure 2:**
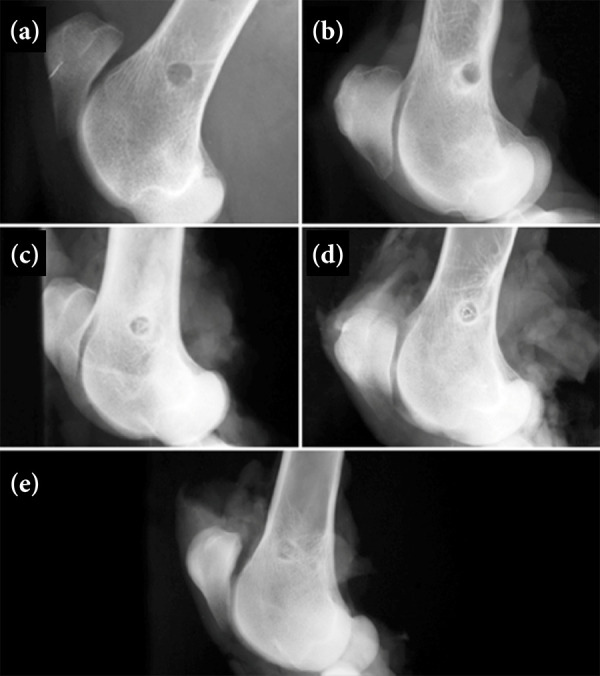
Mediolateral radiographic views of the right distal femur in sheep that received interference screws composed of poly-D, L-lactic acid (PDLLA), **(a)** immediately after surgery, and at **(b)** one month, **(c)** four months, **(d)** seven and a half months and **(e)** 18 months postoperatively. From **(c)** four months a triangular-shaped radiopaque image is visible in the center of the circular radiolucent area, which has become less distinct and resembles bone **(e)** 18 months after surgery.

**Figure 3:**
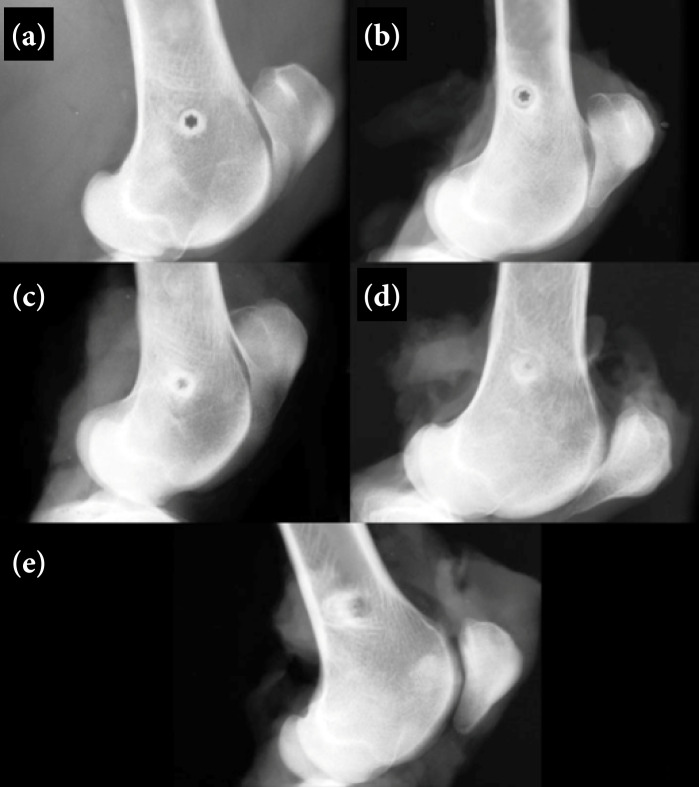
Mediolateral radiographic views of the left distal femur in sheep that received interference screws composed of poly-D, L-lactic acid with beta-tricalcium phosphate (PDLLA/β-TCP), **(a)** immediately after surgery, and **(b)** in one month, **(c)** four months, **(d)** seven and a half months and **(e)** 18 months postoperatively. Note a circular radiopaque area with a six-pointed star-shaped radiolucent central region corresponding to the shape of the screw **(a)** in the immediate postoperative period, and the presence of a radiolucent line between screw and bone **(b)** one month after surgery. In subsequent periods, (c, d) there was progressive central radiopacity with loss of definition of the central screw hole, and **(e)** at 18 months, a less radiopaque area was noted at the screw site.

After the isolated reports of the three evaluators, a consensus was formed, which is described ahead, together with the representative radiographic images (Figs. 2 and 3).

Only the mediolateral projection was used for analysis, due to poor visualization of the screw in the craniocaudal projection. In the immediate postoperative period, a radiolucent circular area corresponding to the screw insertion site was observed in the distal femurs that received a PDLLA screw (Fig. 2a). A similar X-ray image was examined one month after surgery, and an area of sclerosis was observed around the radiolucent circular area (Fig. 2b). At four months postoperatively, a radiopaque triangular image was found in the center of the circular radiolucent area (Fig. 2c). After seven and a half months postoperatively, the radiographic appearance was similar, but the radiopacity increased in the center of the circular radiolucent area (Fig. 2d). At 18 months postoperatively, there was a less distinct radiolucent area with the presence of central radiopacity (Fig. 2e).

In the immediate postoperative period, the femurs that received the PDLLA/β-TCP screw had a circular radiopaque area with a central radiolucent region in the shape of a six-pointed star, corresponding to the design of the central cannula (Fig. 3a). One month after the operation, a radiolucency between the screw and the bone was identified, with an area of sclerosis around the screw (Fig. 3b). At four months postoperatively, radiopacity was maintained around the screw, but the central region became less radiolucent with the loss of the six-pointed star shape (Fig. 3c). Seven and a half months after surgery, the radiopacity around the screw was less distinct, and the central radiopaque area increased, so that the six-pointed star could no longer be identified (Fig. 3d). At 18 months postoperatively, radiopacity around the screw, a radiolucent circular area appears at the screw site, and a central radiopaque area similar in appearance to the adjacent bone was observed (Fig. 3e).

### CT scans

**Figure 4:**
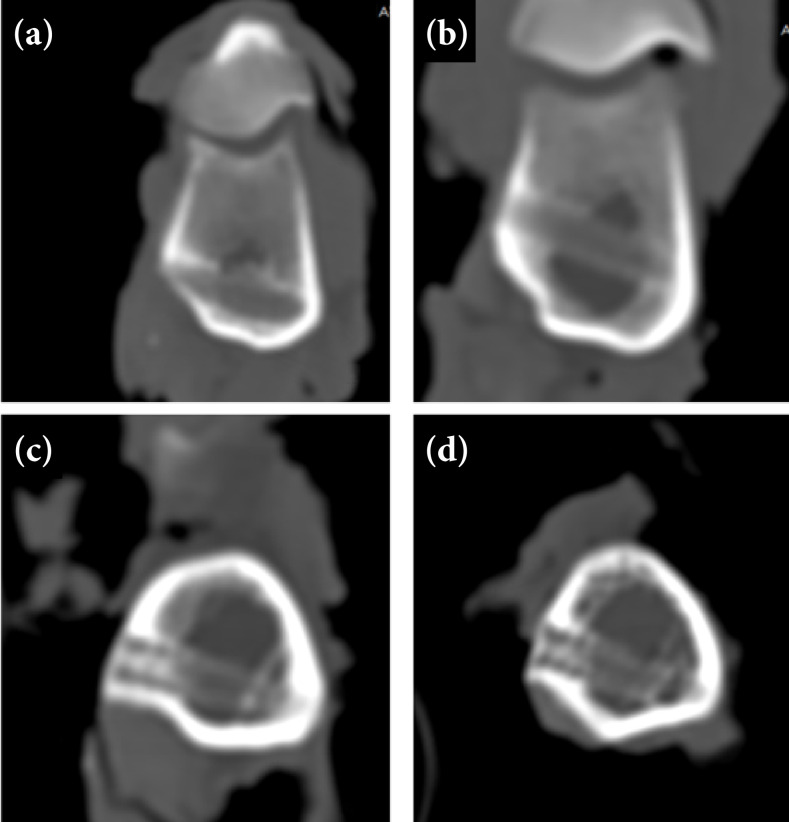
Transverse computed tomography images of the right distal femur in sheep that received interference screws composed of poly-D, L-lactic acid (PDLLA) at **(a)** one month, **(b)** four months, **(c)** seven and a half months, and **(d)** 18 months postoperatively. **(a)** One month after surgery, the interference screw insertion site was hypodense and its contour could not be identified, and greater density adjacent to the screw in the cortical bone was visible **(b)** four months after surgery. Density increased more in the central axis and cortical region in (c, d) the other evaluation periods.

At one month postoperatively, the insertion site of the PDLLA interference screw was hypodense, and its contour could not be identified (Fig. 4a). At four months after surgery, a greater density adjacent to the screw in the cortical bone was visible (Fig. 4b). At seven and a half months postoperatively, the screw contour remained hypodense, but an increase in density along the central axis was observed (Fig 4c). A similar appearance was seen 18 months after surgery, but loss of definition of the screw contour was observed (Fig. 4d).

**Figure 5:**
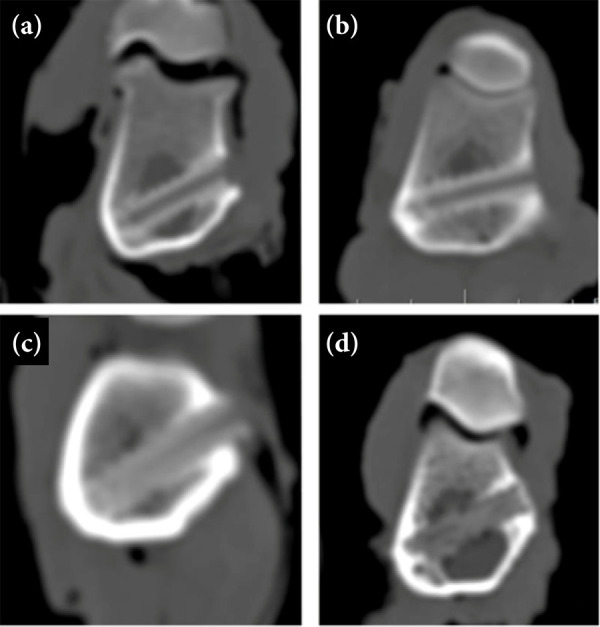
Transverse computed tomography images of the left distal femur in sheep that received interference screws composed of poly-D, L-lactic acid with beta-tricalcium phosphate (PDLLA/β-TCP) at **(a)** one month, **(b)** four months, **(c)** seven and a half months and **(d)** 18 months postoperatively. The screw contour was clearly visible at **(a)** one month after surgery, and greater density on the central axis was seen at **(b)** four months postoperatively. The screw contour was less evident in (c, d) the other evaluation periods, and the screw insertion site had a density similar to the one of the medullary bone at **(d)** 18 months postoperatively.

At one month postoperatively, the contour of the PDLLA/β-TCP screw was clearly visible, and its threads were identified (Fig. 5a). The screw was hyperdense in relation to the medullary bone, but hypodense in relation to the cortical bone. At four months after surgery, screw contour was less evident than in the previous assessment, and greater density in the central axis was observed (Fig. 5b). Seven and a half months after the operation, there was loss of definition of the screw (Fig. 5c). At 18 months after the operation, the screw body had a density similar to the medullary bone (Fig. 5d), with the end of the screw being hyperdense, in the region of the cortical bone.

The Hounsfield unit values of PDLLA screws showed significant differences at one and four months after surgery compared to 18 months after surgery, which had higher density. In all evaluation periods, Hounsfield unit values differed significantly between PDLLA and PDLLA/β-TCP. However, the density of PDLLA was greater than that of PDLLA/β-TCP only at 18 months postoperatively (Table 1).

**Table 1:** Hounsfield unit (HU) values obtained on computed tomography of screws composed of poly-D, L-lactic acid (PDLLA) or PDLLA with beta-tricalcium phosphate (PDLLA/β-TCP) at **(a)** one, **(b)** four, **(c)** seven and a half and **(d)** 18 months postoperatively.

Evaluation periods (month)	N	PDLLA screws (HU)	PDLLA/β-TCP screws (HU)
1	5	172.08 Bb	416.75 Aa
4	6	175.90 Bb	445.10 Aa
7.5	5	276.33 ABb	508.25 Aa
18	6	318.80 Aa	220.20 Bb
**ANOVA factors**	**F**	**p-value**	
Periods	6.30	0.001	
Screws	61.12	<0.0000001	
Periods*screws	15.16	0.00000153	

N: number of samples in each group; means follow by the same letters, uppercase in columns and lowercase in rows, do not differ by Tukey’s test at 5% probability.

### MicroCT and histology

**Figure 6:**
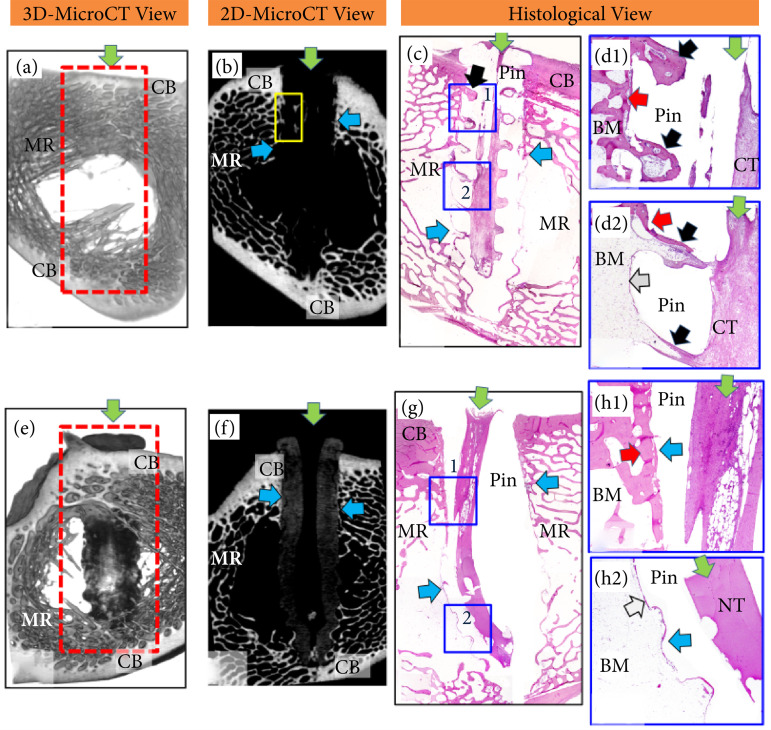
Three- and two-dimensional images obtained from microcomputed tomography, as well as histological sections stained with hematoxylin-eosin, of (a-d) the insertion region of a PDLLA screw and (e-h) a PDLLA/β-TCP screw four months after the surgery. Three- and two-dimensional views: microcomputer tomography. Note the screw insertion site (*red rectangular area*) around the cortical (CB) and medullary (MR) regions and the green arrow indicating the central region of the screw. Note that **(a)** the PDLLA screw is hypodense with small hyperdense areas (yellow rectangular area) in the region of the lateral holes, while **(e)** the PDLLA/β-TCP screw is hyperdense with a hypodense central cavity. Histological sections: the panoramic image of the **(c)** PDLLA screw shows the central screw cavity (green arrow), side holes (black arrow) and threads (blue arrows). Note the change in contour and widening in the region of the PDLLA/β-TCP screw located in the region of the medullary cavity. Note in detail d1, two holes (black arrow) of the PDDLA screw filled with bone tissue and bone marrow, and the thread surface surrounded by bone tissue composed of trabeculae (red arrow). Note in detail d2, the central screw cavity filled with connective tissue (CT) connected to the screw holes (black arrow) filled with medullary tissue (BM), containing threads of bone tissue (red arrow), while the thread surfaces (arrow blue) are covered by a thin connective tissue capsule (grey arrow). In comparison, **(g)** the PDLLA/β-TCP screw showed absence of holes and central cavity (green arrow) mainly filled with fibrous connective tissue / bone marrow in the upper portion (detail h1), while the lower portion was filled with tissue necrosis (NT). Note in detail h1 the presence of bone formation (red arrow) on the surface of the screw threads (blue arrow), located close to the cortical region. Note in detail h2 the presence of a thin fibrous capsule in the screw threads located in the center of the medullary region.

**Figure 7:**
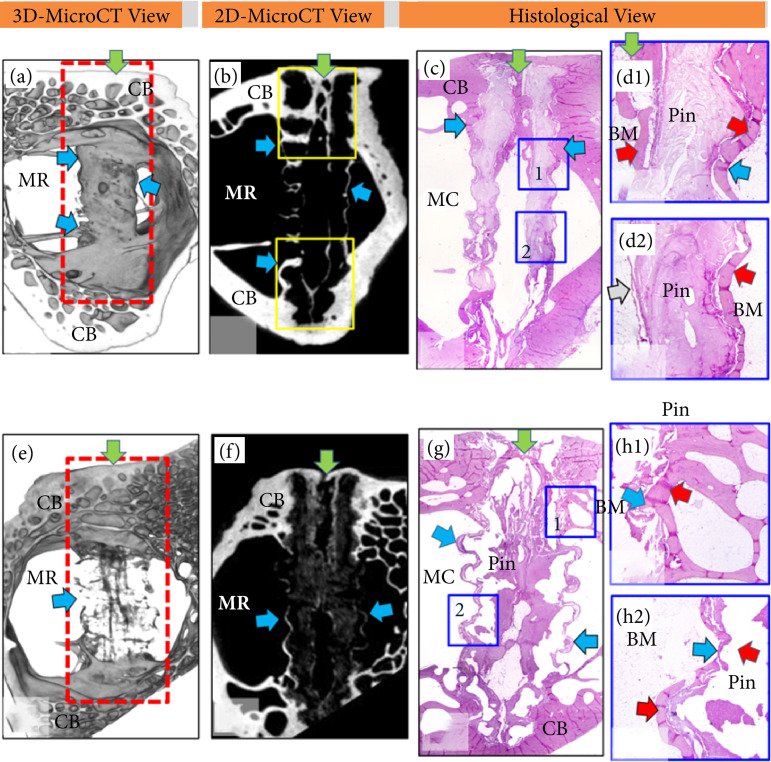
Three- and two-dimensional microcomputer tomography images, as well as hematoxylin-eosin stained histological sections, of the insertion region of (a-d) a PDLLA screw and (e-h) a PDLLA/β-TCP screw 18 months after surgery. Three- and two-dimensional views: microcomputer tomography. Note the screw insertion site (red rectangular area) around the cortical (CB) and medullary (MR) regions and the green arrow indicating the central region of the screw. Note that **(a)** the PDLLA screw is hypodense with hyperdense areas (yellow square areas) in the regions of the lateral holes and in the central cavity of the screw (blue arrows). Implantation of **(e)** PDLLA/β-TCP is less hyperdense than earlier periods with mild central cavity hyperdensity (green arrow). **(e)** The PDLLA/β-TCP screw was less hyperdense compared to previous periods with a mildly hyperdensity central cavity (green arrow). Histological sections: **(c)** panoramic image of the PDLLA screw demonstrates dense bone formation (red arrow in detail d1) around the screw surface and inside the central cavity (green arrow), which made it difficult to remove the PDLLA screw during processing histological. Thus, an amorphous structure in the region (Pin) of the PDLLA screw can be seen. Detail d2 shows the portion of the screw located in the center of the medullary region of the femur. Note the central cavity of the screw lined with a thin layer of connective tissue (grey arrow) and filled with medullary bone tissue (BM). Comparatively, the PDLLA/β-TCP screw presented widening in the medullary cavity region of the femur. Note in detail h1 that the screw surface (blue arrow) was completely covered by bone tissue (red arrow) in the region close to the cortical bone, while in detail h2 the screw was covered by fibrous capsule (red arrow) in the center of the bone marrow.

Representative two-dimensional and three-dimensional microCT images and the corresponding histological sections of the right femur that received PDLLA and the left femur that received PDLLA/β-TCP screws are shown in Figs. 6 and 7.

### One month after surgery

MicroCT images revealed hypodense PDLLA screw and hyperdense PDLLA/β-TCP screw. Histologically, trabecular bone formation began between the screw thread and the areas of bone fragment resorption. PDLLA screw holes were filled with bone debris and connective tissue. Dense connective tissue occurred in the screw head. Intense fibroplasia was detected on the thread surfaces of both screws four months after surgery (Fig. 6): the PDLLA screw could not be seen on microCT images due to its hypodense characteristic (Figs. 6a and 6b), while the screw PDLLA/β-TCP was hyperdense. In addition, an increase in the PDLLA/β-TCP screw was observed in the medullary region of the bone (Figs. 6e and 6f). Histologically, in the regions of the cortical bone and in the regions closest to them, the thread contours of the PDLLA (Figs. 6c and 6d1) and PDLLA/β-TCP (Figs. 6g and 6h1) screws were easily observable due to bony tissue deposition on its surface. However, the threads located in the medullary region of the bone were surrounded by a thin layer of connective tissue and rarely by bone tissue (Fig. 6h2). The central cavity and upper lateral holes of the PDLLA screws were filled with compact bone tissue and the remainder with medullary bone and connective tissue (Fig. 6 c-d1-d2). In contrast, the PDLLA/β-TCP screw (Fig. 6g) showed no lateral holes, but a central cavity (green arrow) filled mainly by fibrous connective tissue and bone formation in the upper portion (Fig. 6h1), with bone formation being more evident in cortical portion and discrete in the medullary portion (Figs. 6g, 6h1 and 6h2).

### Seven and a half months after surgery

The characteristic of hypodensity was maintained in the PDLLA screws, while the PDLLA/β-TCP screws were hyperdense. The PDLLA/β-TCP screws were dilated with a reduced central cavity. Histologically, the holes in the PDDLA screws formed a connection between the screw’s central cavity and the surrounding tissues (bone and bone marrow). The central cavity of the PDLLA/β-TCP screws was isolated with bone formation, localized only in the cortical region. The PDLLA and PDLLA/β-TCP screw threads were both covered with bone tissue or thin fibrous capsule.

### Eighteen months after surgery

PDLLA screws were visible on microCT images due to their hyperdense characteristic (Figs. 7a and 7b). PDLLA/β-TCP screws showed reduced density, or hypodensity (Fig. 7f), indicating degradation over time. The PDLLA screws were surrounded by bone tissue, showing good osseointegration–this was very evident on the surface of the screw threads (Figs. 7a and 7b). Histologically, the holes of the PDLLA screws allowed their central cavity to be filled with tissue characteristic of the region (bone and bone marrow), maintaining the structure of the PDLLA screw (Fig. 7c). Due to the presence of β-TCP, PDLLA/β-TCP screws absorb fluid and expand, thus reducing the central screw cavity. Unlike the PDLLA screw, the PDLLA/β-TCP screw was degraded or absent, being replaced by predominantly soft tissue (connective tissue and bone marrow) and rarely by bone tissue (compare Figs. 7g, 7h1 and 7h2 with Figs. 7c, 7d1 and 7d2).

## Discussion

This study evaluated two bioabsorbable interference screws that had PDLLA in the composition and found that addition of β-TCP with PDLLA favored screw degradation. Both screws had 70% L-lactide and 30% D L-lactide, similar to the proportion used in plates and screws for anchors^9^, maxillofacial surgery^10^, and some interference screws^11^^,^ 12. The selection and distribution of stereoisomers within polymer chains influence the thermal, mechanical and biodegradation characteristics of PLA^3^^,^ 13. In line with this, a study of lactide stereo-copolymers with different L/D ratios found that the ratios of 90/10 and 85/15 have good strength, but very slow hydrolytic degradation^14^. On the other hand, although the screws used in our study were similar in size and shape, the PDLLA screws had seven axial holes. Despite the importance of screw holes for bone formation along them^11^, a mechanical study showed that 21-hole PDLLA screws have lower stiffness than nine-hole screws, although all screw models tested showed safe strength, since the insertion torque of the screws was always lower than the fracture torque^15^.

There were no trans- or post-operative complications observed after insertion of PDLLA or PDLLA/β-TCP screws in the present study. However, the use of bioabsorbable interference screws in human patients has been associated with foreign body reactions, synovitis, tunnel enlargement, osteolysis, intraosseous cyst formation, effusion, systemic allergic response, intra-articular foreign bodies, as well as breakage during insertion or failure to hold the graft^1^^,^ 2 ^,^ 9 ^,^ 16 ^,^ 17. A wrench that fit the entire length of the screw was used in the present study since screw breakage can occur if a wrench is not placed to extend to the end of the screw. Screw failure during anterior cruciate ligament reconstruction has been related to an insertion device in a case series in human patients^18^.

The radiographic evaluation showed that the bone reaction around and inside varied between screws, as PDDLA screws were visualized four months after surgery and PDLLA/β-TCP were detected immediately after surgery due to the presence of β-TCP. The assessment of degradation of absorbable screws can use radiography, magnetic resonance image (MRI), and CT, with the CT being most commonly used to assess the resorption of screws and bone growth provided at the site^19^. When using CT in the present study, it showed that the PDLLA screw was hypodense at all times of the evaluation, but with a progressive increase in density along the central axis, while the PDLLA/β-TCP was initially hyperdense and progressively lost this characteristic. These findings also corresponded to the Hounsfield unit values obtained in the CT scans, as at 18 months after surgery the PDDLA screws had higher Hounsfield unit values, while the PDLLA/β-TCP screw had lower Hounsfield unit values compared to the initial evaluations. In turn, PDLLA cannulated interference screws used for fixation of the anterior cruciate ligament graft in human patients were visible on MRI and CT at six to eight months after surgery, but less distinct with centralized growth of connective tissue at 12 to 16 months postoperatively^20^.

In histology and micro-CT analysis, both screws showed reduced production of bone tissue at four months after surgery, which was more evident in contact with the cortical bone. Since PLLA remains *in vivo* for up to five years and it is completely reabsorbed at seven years^19^, faster degradation has been produced by combining different amounts of D and L lactide^3^ as used in the present study. Although PDLLA stereo isomers present faster degradation, a greater inflammatory response may occur^9^. However, this was not evident during histological analysis of the current study for PDDLA or PDLLA/β-TCP screws. Also, in a study that assessed PLLA screws combined with hydroxyapatite or ß-TCP implanted in sheep femur, no signs of inflammation or neutrophil presence were found on the postoperative days 42, 50, 57 or 84^21^.

At seven and a half months postoperatively, histologically the PDLLA/β-TCP screws showed reduction in the central cavity, while the PDLLA screws showed bone filling through the lateral holes to the central cannula. In general, bioabsorbable polymers are surrounded by a fibrous layer, whereas bioactive ceramics develop a bone-like apatite layer^1^. A decrease of PDLLA/β-TCP screw density was observed by means of microCT at 18 months after surgery, which was histologically degraded or absent. However, screws were replaced by connective tissue and bone marrow, in the medullary region, and by dense bone tissue only in the cortical region.

We can assess that 18 months is an insufficient time for bone production at the PDLLA/β-TCP screw site in this study in sheep. Difference between organic reactions in animals and humans should be considered, since a systematic review of degradation and performance of PLGA/β-TCP interference screws in human patients showed 88% of loss of original volume around 30 months after surgery, with osteoconductivity promoting in 63% of cases^17^. Another situation is that the amount of β-TCP can be increased, as a study of the performance of PLLA/β-TCP materials found that increasing the percentage of ß-TCP in relation to the lactic acid polymer induced the synthesis so dose-dependent extracellular bone matrix, as well as proliferation of osteogenic cells^22^. On the other hand, high concentrations of ceramic material can reduce mechanical properties^23^. Furthermore, in the present study, the PDLLA/β-TCP screw did not contain lateral perforations. Histological evaluation of PDLLA interference screws inserted in the proximal tibia in sheep identified connective tissue growth without signs of ossification in non-drilled screws and cancellous bone in all holes and core in drilled screws, 24 weeks after the operation^11^. Likewise, the results of the current study 18 months after surgery showed that the PDLLA screws remained hypodense, but surrounded by bone tissue on microCT, and histologically the holes allowed filling of the central cavity with bone tissue. These findings reinforce the importance of screw geometry, not just material composition^1^^,^ 11.

The degradation of the PDLLA screw in the present study was not histologically evident at 18 months. However, in a study of human patients who received PDDLA screws [made from an amorphous stereopolymer, poly- (l-co-d, l-lactide) 70:30] for fixation of the anterior cruciate ligament graft, the site densities MRI screws were comparable with surrounding bone density at 22 months postoperatively^14^. Thus, the major limitation of this study was that 18 months of evaluation were not sufficient to determine the degradation time point of the PDLLA screw. Another limitation was that the performance of the screws to secure ligament/graft in bone tunnels were not assessed. Therefore, future studies should further examine these issues.

## Conclusion

The inclusion of β-TCP favors screw degradation since the PDLLA/β-TCP screws evidenced a more intense degradation process than the PDLLA screws at the last evaluation. PDLLA screws showed higher bone production, evident around the screw thread, inside the lateral perforations, and in the central canal, whereas the PDLLA/β-TCP screws presented less bone tissue at the implantation site.
